# Methylation-Associated Gene Silencing of *RARB* in Areca Carcinogens Induced Mouse Oral Squamous Cell Carcinoma

**DOI:** 10.1155/2014/378358

**Published:** 2014-08-17

**Authors:** Zi-Lun Lai, Yung-An Tsou, Shin-Ru Fan, Ming-Hsui Tsai, Hsiao-Ling Chen, Nai-Wen Chang, Ju-Chien Cheng, Chuan-Mu Chen

**Affiliations:** ^1^Department of Life Sciences, Agricultural Biotechnology Center, National Chung Hsing University, No. 250 Kao-Kuang Road, Taichung 402, Taiwan; ^2^Department of Otolaryngology Head and Neck Surgery, China Medical University, Taichung 404, Taiwan; ^3^School of Medicine, College of Medicine, China Medical University, Taichung 404, Taiwan; ^4^Department of Medical Laboratory Science and Biotechnology, China Medical University, No. 91 Hsueh-Shih Road, Taichung 404, Taiwan; ^5^Department of Bioresources, Da-Yeh University, Changhwa 515, Taiwan; ^6^Department of Biochemistry, College of Medicine, China Medical University, Taichung 404, Taiwan; ^7^Rong Hsing Research Center for Translational Medicine, iEGG Center, National Chung Hsing University, Taichung 402, Taiwan

## Abstract

Regarding oral squamous cell carcinoma (OSCC) development, chewing areca is known to be a strong risk factor in many Asian cultures. Therefore, we established an OSCC induced mouse model by 4-nitroquinoline-1-oxide (4-NQO), or arecoline, or both treatments, respectively. These are the main two components of the areca nut that could increase the occurrence of OSCC. We examined the effects with the noncommercial MCGI (mouse CpG islands) microarray for genome-wide screening the DNA methylation aberrant in induced OSCC mice. The microarray results showed 34 hypermethylated genes in 4-NQO plus arecoline induced OSCC mice tongue tissues. The examinations also used methylation-specific polymerase chain reaction (MS-PCR) and bisulfite sequencing to realize the methylation pattern in collected mouse tongue tissues and human OSCC cell lines of different grades, respectively. These results showed that retinoic acid receptor *β* (*RARB*) was indicated in hypermethylation at the promoter region and the loss of expression during cancer development. According to the results of real-time PCR, it was shown that *de novo* DNA methyltransferases were involved in gene epigenetic alternations of OSCC. Collectively, our results showed that *RARB* hypermethylation was involved in the areca-associated oral carcinogenesis.

## 1. Introduction

Throughout the world, oral squamous cell carcinoma (OSCC) is one of the most common types of cancers. It has a high cure rate for small primary tumors and involves the development of second primary tumors and the long-term survival rate is <60% [1]. Furthermore, in Taiwan, according to statistics from the Department of Health, Executive Yuan, Taiwan, OSCC ranks as fourth among the ten leading causes of cancer among males and is the fourth leading cancer in the male population and the number of deaths increases every year [2]. The main risk factor for developing OSCC is chewing areca, especially in many Asian cultures.

In a clinical study, the incidence of oral cancer was elevated 28 times for betel quid users as compared to nonbetel quid users [3]. Cigarette smoking has synergistic effect with areca chewing, and such users have an 89 times higher incidence rate than nonusers. If one has the habit of drinking, smoking, and betel quid chewing combined, there will be a 123 times higher incidence rate of having oral cancer than those average individuals in the general population that are nonusers. There is the longitudinal cohort study on the alcohol, betel quid, and smoking, to the oral cancer risk. The betel quid partook the significant higher hazard risk to the oral carcinogenesis [4]. The most tumorigenic part of betel quid is the* Piper longum* L. and the calcium hydroxide (slaked lime) which will cause the oral cavity to develop into an alkaline condition which will promote the tumorigenic effect of the Safrole in the* Piper longum* L. The fibers of areca also cause oral mucosa damage and increased mucosa to be exposed to the tumorigenic material in the betel quid. In a clinical survey, oral cancer with areca chewing had far more incidence of oral submucosa fibrosis and erythroplakia; furthermore, the pathologic findings also show severe hyperkeratosis, caries, and gingivitis as compared to nonareca users. The oral cancer patients who had the habit of betel quid chewing were also found to have a higher percentage of dysplastic change surrounding the tumor margin, and skip cancer lesion was frequently noted in the upper aerodigestive tract (tongue, hypopharynx, and esophagus). The condemned mucosa even reached the esophagus. In an average clinical survey, 18% of cases were found to have esophagus cancer diagnosed at the same time when oral cancer presented [5]. Synchronous double cancer (second primary cancer) in the upper aerodigestive tract is frequently noted [6].

There was a stimulating effect when areca nut is chewed along with betel leaf [7]. Furthermore, in Chiang et al. [8], they used areca nut extract (ANE) and saliva-reacted ANE (sANE) to treat three oral carcinoma cell lines, KB (epidermoid carcinoma), SAS (tongue carcinoma), and Ca9-22 (gingival carcinoma). The higher cytotoxic effects involving cell morphologic changes and upregulation of inflammatory signaling in mRNA expression levels were observed in these treatments. In addition, arecoline is the major alkaloid in areca nut extracts and betel quid. It is the primary active ingredient responsible for the central nervous system simulation that is roughly comparable to that of nicotine, which has a similar chemical structure [9–12]. There is also another carcinogen, 4-nitroquinoline 1-oxide (4-NQO), which effectively induces oral and esophageal cancers that closely resemble early human lesions in mice and rats [13, 14]. In this study, we followed Chang et al. [15] who established an effective mouse model of oral cancer and used this model to identify potential markers of oral tumor progression by utilizing a noncommercial methylation microarray.

The promoter hypermethylation now has a key role for research in the area of human multistage carcinogenesis. Silencing of certain tumor suppressor genes may occur in the absence of genetic change, via aberrant methylation of CpG islands [16–18]. OSCC is believed to arise through the accumulation of numerous genetic and epigenetic alterations [19–21]. There are several methods to determine whether promoter methylation has been developed including combination of bisulfite restriction assay (COBRA) [22], genomic bisulfate sequencing [23], methylation-specific PCR (MS-PCR) [24], and microarray-based methylation analysis [25]. Methylation microarray is a high throughput tool for genome-wide methylation analysis [26–31]. To identify and characterize potential targets for treating oral cancer, a genome-wide approach was taken to quantitatively measure genomic alterations in OSCC [32, 33]. Consequently, we used home-made mouse CpG island microarray to understand aberrant methylation profile during OSCC tumorigenesis in this study and validated the methylation status by MS-PCR, bisulfite sequencing, and real-time PCR.

In many previous studies, retinoid acid suppresses carcinogenesis and inhibits the growth of human head and neck squamous cell carcinoma (HNSCC) [34, 35]. Loss of retinoids and their receptors has been associated with malignant progression in HNSCC [36]. Their receptors (RAR) are central regulators to the normal growth and differentiation of a variety of epithelial cells. RAR changes have been associated with cell immortalization, and re-expression of* RAR-beta* (*RARB*) leads to growth inhibition in some circumstances [19]. Loss of* RARB* expression is associated with a change in proliferative life span potential from mortality to immortality in HNSCC [37–39]. The promoter hypermethylation of* RARB* could inhibit the gene expression when added to the methylation inhibitor and deacetylation inhibitor like 5′-aza-2′-deoxycytidine (5-aza-dC) and trichostatin A (TSA) which could recover the gene expression and inhibit tumor cells growth [36, 39].

In the present study, we investigated the role of* RARB* hypermethylation of CpG islands in OSCC mouse model and its association with* RARB* expression in human oral cancer cell lines. In addition, we examined whether the repression of* RARB* transcription could be reversed by 5-aza-dC in human oral cancer cell lines, and finally, we evaluated the three main DNA methyltransferases that were involved in* RARB* hypermethylation.

## 2. Materials and Methods

### 2.1. Mouse Model for Oral Cancer

The mouse model development was modified as highlighted by Chang et al. [15, 40]. Briefly, the OSCC model was established by treating arecoline (Sigma, St. Louis, MO), as well as in combination with 4-NQO (Fluka, St. Louis, MO) in 4-5 week age old of C57BL/6JNarl male mice. The conditions for OSCC formation are 500 *μ*g/mL arecoline (A), 200 *μ*g/mL 4-NQO (N), and 4-NQO (200 *μ*g/mL) combined with arecoline (500 *μ*g/mL) (NA) in the drinking water for 8 weeks. The drinking water was changed every day, and mice were allowed access to the drinking water at all times while receiving treatment. After the treatment, the drinking water was changed to ddH_2_O and mice were sacrificed at week 8, 12, 14, 18, 20, 26, and 28, respectively. The tongues were collected and classified into tumor parts (T) and nontumor parts (NT) for mouse CpG island microarray analysis.

### 2.2. Cell Culture and 5-aza-dC Treatment

Normal human oral keratinocytes (NHOK) were cultured in Keratinocyte Growth Medium (KGM, GIBCO, CA, USA). Oral cancer cell lines, DOK, OC2, and Ca9-22, were cultured in DMEM (GIBCO, CA, USA). HSC3 and TW2.6 were cultured in DMEM-F12 (GIBCO, CA, USA). All cells supplemented with 10% fetal calf serum and 1% penicillin-streptomycin and cultured at 37°C with 5% CO_2_. Oral cancer cells were treated by 5-aza-dC at 2 *μ*M to reverse the methylation status as described in [36, 41].

### 2.3. DNA and RNA Extraction

The genomic DNA extraction was conducted as noted in our previous report [42]. We used collected nontumor parts (NT) and tumor parts (T) of tongues for DNA and RNA extractions.

### 2.4. RT-PCR and Real-Time PCR

Total RNA was prepared using the TRI REAGENT (Invitrogen, CA, USA). One microgram of total RNA was treated with 10 units of RQ1 RNase-Free DNase (Prome ga, WI, USA) and extracted with phenol-chloroform. DNase that treated total RNA (1 *μ*g) was reverse transcribed with the ImProm II Reverse Transcription System (Promega, WI, USA). For RT-PCR amplification was performed with 2720 thermal cycler (Applied Biosystems Inc., CA, USA) and Real-Time PCR amplification was performed with Rotor-Gene 6000 (Corbett, CA, USA). The amplification of RT-PCR was repeated for 28 cycles as follows: 95°C, 30 sec for denature of the annealing temperature depending on the pair of gene specific primer sets (Table 1) for 30 sec and 72°C, 30 sec for extension. PCR reactions were performed in triplicate and the transcription level was normalized with the* GAPDH*. For Real-Time PCR, the calculated gene expression fold from CT value was performed according to the previously mentioned study, with a *P* value of less than 0.05 exhibiting an obviously significant difference.

### 2.5. Preparation of Mouse CpG Island Microarray and Amplicon Generation

The mouse CpG island microarray was based on previously described human CpG island microarrays [43–46]. A total 2,304 mouse CpG islands library (mCGI) clones were spotted on UltraGAPS Coated Slides (Corning, MA, USA) by the BioDot AD1500 (BIODOT, CA, USA). The amplicons for methylation analysis were prepared as previously described [47, 48].

### 2.6. Microarray Hybridization and Data Analysis

The purified amplicons (5 *μ*g) were conducted using the BioPrime DNA labeling system (Invitrogen, CA, USA). Cyanine 5-ddUTP (Cy5-ddUTP) and Cyanine 3-ddUTP (Cy3-ddUTP) (Perkin-Elmer Life Sciences, NJ, USA) fluorescent dyes were coupled to tumor (T) and normal (NT) amplicons, respectively, and cohybridized to the microarray panel. The combined tumor/normal control pair, with 8 *μ*g DNA, more than 180 pmol Cy5, and 150 pmol Cy3, would give strong hybridization signals. The hybridization of 4,608 spots is carried out under a 24 × 50 mm cover glass sealed tightly within a moistened hybridization chamber, GeneMachines HybChambers (Genomic Solutions, MI, USA), in a 65°C water bath from 12 to 16 h. The posthybridization washing steps are essentially those described by UltraGAPS Coated Slides instruction Manual. The hybridized slides were scanned with the GenePix 4000B scanner (Axon, CA, USA) and the acquired images were analyzed with the software GenePix Pro 4.0 (Axon, CA, USA). The microarray data was analyzed as described previously [43–46, 48, 49]. Briefly, the Cy5/Cy3 ratio and the hybridization intensity from the tumor amplicons to the hybridization intensity from the normal amplicons, from each image, are normally guided by both the average global Cy5/Cy3 ratio from each image and the Cy5/Cy3 ratios from 9 internal controls (clones without restriction cutting sites whose copy numbers remain the same in tumors and normal samples). Yellow spots (normalized Cy5/Cy3 = 1) represent equal amounts of bound DNA from each amplicon, indicating no methylation differences between tumor (T) and nontumor (NT) genomes. The analyzed data were using hierarchical clustering to classify the relationships of all genes between collected T and NT samples. A hierarchical clustering algorithm was used to investigate relationships among tumor samples. The complete linkage and the dissimilarity measure (1 minus the Pearson correlation coefficient of the log-adjusted Cy5 : Cy3 ratios) were used for the analysis. The resultant dendrogram showed linked closely related colorectal tumors into a phylogenetic tree whose branch lengths represented the degree of similarity between these tumors.

### 2.7. Bisulfite Sequencing and Methylation-Specific PCR (MS-PCR) for Methylation Status Analysis

Genomic DNA (~0.5 *μ*g) was treated with sodium bisulfite according to the manufacture's recommendations (EZ DNA Methylation Kit; Zymo Research, CA, USA). All selected genes methylation statuses were examined by methylation-specific PCR (MS-PCR) and sodium bisulfite genomic sequencing. The PCR reaction was as follows: 95°C for 5 min, followed by 45 cycles of 95°C, 30 sec, Tm for 30 sec (Table 1), 72°C for 45 sec and ended with an extension of 72°C for 5 min and quick chill to 4°C on a Geneamp2400 PCR system (Applied Biosystems, CA, USA). For bisulfate sequencing analysis, each PCR product was subcloned into the pGEM-T Easy Vector (Promega, WI, USA) and performed 5–10 clones in each selection, respectively. Each colony was sequenced using the BigDye Terminator v3.1 Cycle Sequencing Kit and the automated ABI PRISM 3100 Genetic Analyzed (Applied Biosystems, CA, USA).

### 2.8. Statistical Analysis

Statistical analysis was performed using *t*-test to examine the association between NHOK and other cell lines. One-sided testing was used to calculate the *P*, and *P* < 0.05 was considered statistically significant.

## 3. Results

### 3.1. OSCC Mouse Model Induced by Arecoline and 4-NQO

**Figure 1:**
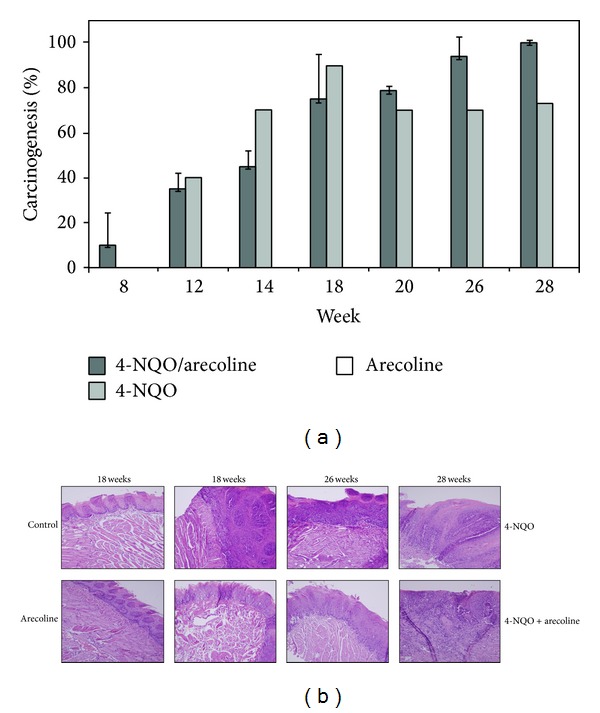
The progression of mouse model development for OSCC. (a) The ratio of carcinogenesis in mouse OSCC model. There were three treatments, 4-NQO/arecoline, 4-NQO, and arecoline. Mice were sacrificed at weeks 8, 12, 14, 18, 20, 26, and 28, respectively. The scoring criteria for mouse OSCC model are described in Section 2. (b) OSCC tongue tissues with tumors were excised, fixed, embedded, and sectioned for H&E staining. The mice that were treated with 4-NQO + arecoline would induce more serious OSCC formation than 4-NQO only and arecoline only. The order of severity was followed the time of treatment.

To evaluate the efficiency of mouse model involving cotreating with arecoline and 4-NQO that mimic the etiology for OSCC tumor growth, the percentage of mice exhibiting carcinogenesis was calculated in Figure 1(a). Tumor development was assessed when treated with 4-NQO (N) and 4-NQO plus arecoline (NA) but not in arecoline. Mice were also sacrificed at 18, 26, and 28 weeks, and tongues with tumors were excised, fixed, embedded, and sectioned for H&E staining (Figure 1(b)). The H&E staining also showed that the tumor progresses were dealing with time and according to the treatments. According to the results, the incidence of tongue carcinogenesis in NA group was significantly higher than N group and arecoline group. Taken together, the treatment of NA at week 28 was much more serious than other weeks. These results suggest that arecoline promotes 4-NQO carcinogenesis in damaged oral epithelia cells.

### 3.2. Identification of Hypermethylation Genes from OSCC Tissues by CpG Island Microarray

**Figure 2:**
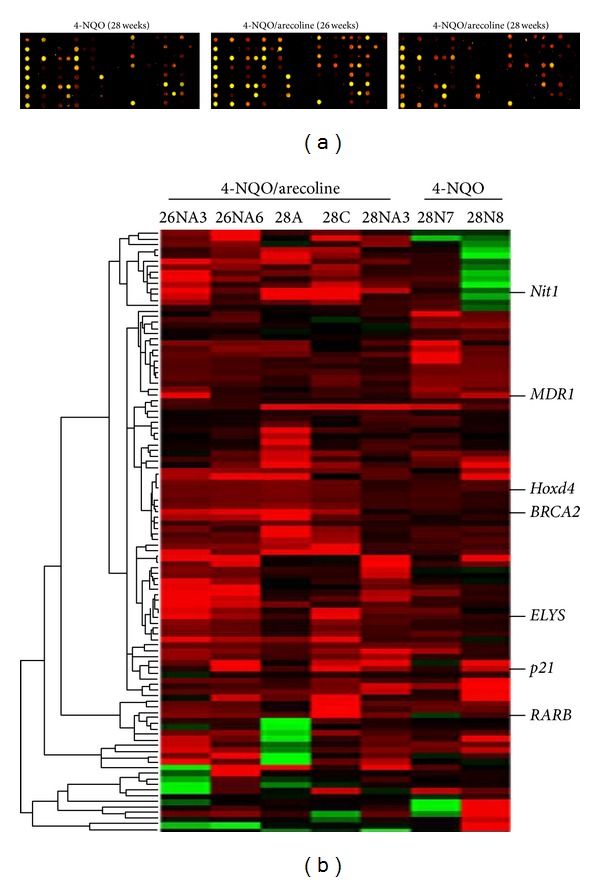
Presenting of MCGI microarray hybridization results and hierarchical clustering of methylation data of OSCC model mice. (a) MCGI microarray hybridization panel contained 4,608 duplicated CpG island tags. The expanded hybridization views showed the usefulness of the MCGI microarrays cohybridized with fluorescently labeled T (tumor part) and NT (nontumor part) with N at 28 weeks and NA at 26 and 28 weeks. Spots hybridized predominantly with tumor amplicon but not with normal amplicon would appear red and are indicative of hypermethylated CpG island loci present in the tumor genome. (b) Hierarchical clustering of N (4-NQO) and NA (4-NQO/arecoline) samples. At the top lists the 5 NA and 2 N studied. The row corresponds to each of 109 CpG island loci selected for methylation analysis. CpG islands (the normalized Cy5 : Cy3 ratios are ≧2) are those with hypermethylation in tumor DNA. The selected hypermethylated candidates were showed on the right sides. They are described in greater detail in Table 2.

**Figure 3:**
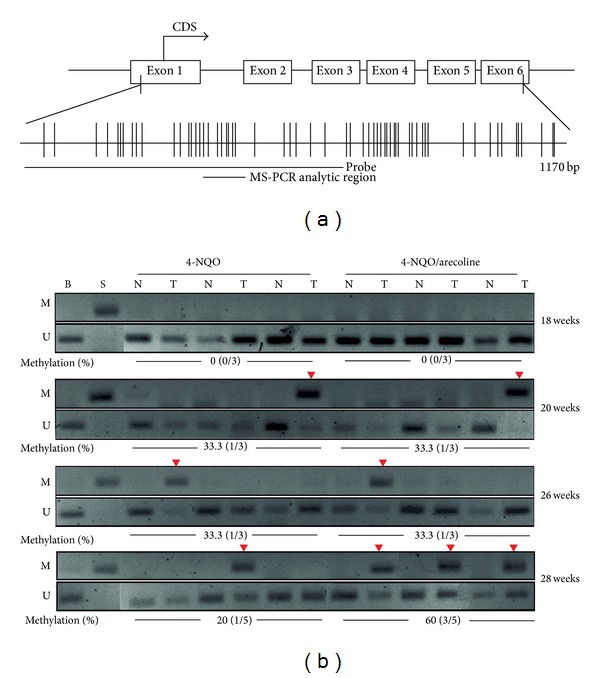
Methylation status of* RARB* gene in mouse OSCC model. (a) The CpG island diagram of* RARB* gene. The hybridized probe used to microarray located from exon one to exon three. We designed the MS-PCR primer sets located within this region. (b) The designed MS-PCR analysis results in 4-NQO and 4-NQO/arecoline at 18, 20, 26, and 28 weeks, respectively. The* RARB* gene showed hypermethylation (60%) in 4-NQO/arecoline at 28 week. M: methylated set, U: unmethylated set, B: blood DNA for methylation negative control, S:* Sss*I treated DNA for methylation positive control, and %methylation: the percentages of methylation. The red inverted triangle showed that the methylated* RARB* gene could be amplified.

**Figure 4:**
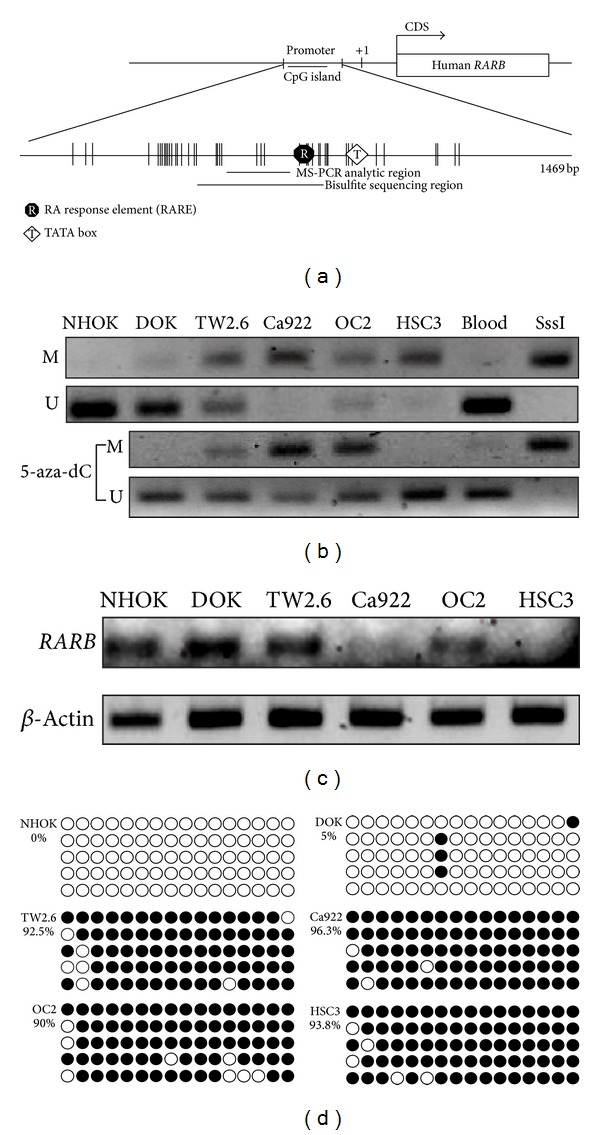
Methylation status in human oral cancer cell lines of* RARB*. (a) The schematic for CpG island presentation of* RARB*. There was a CpG island located in promoter region. We designed the MS-PCR and bisulfite sequencing primer sets in this region. (b) The* RARB* methylation status in different human oral cancer cell lines. The cell lines were also treated with 5′-aza-dC. The methylation status was recovered because of 5′-aza-dC treatment. (c) The* RARB* mRNA expression in different human oral cancer cell lines. The* RARB* was not expressed in Ca922, OC2, and HSC3 obviously. (d) The* RARB* bisulfite sequencing in different human oral cancer cell lines. There were more methylated* RARB* in TW2.6, Ca922, OC2, and HSC3 than in NHOK and DOK. The hollow circle is the unmethylated CpG site and the full circle is the methylated CpG site.

According to the tumor progression percentages and H&E staining results, we selected the tongues tissues to be targets from OSCC mouse model with N and NA at 26 and 28 weeks for MCGI microarray screening.  Figure 2(a) depicts representative data from the OSCC study. The expanded hybridization views showed the usefulness of the MCGI microarrays cohybridized with fluorescently labeled T (tumor part) and NT (nontumor part) with N at week 28 and NA at weeks 26 and 28, respectively. Spots hybridized predominantly with tumor amplicon, but not with nontumor amplicon, would appear red and are indicative of hypermethylated CpG island loci, present in the tumor genome. The hybridization results showed that treatment with NA at week 28 represented much more spots that were obviously more hypermethylated than other treatments. Yellow spots (Cy5 : Cy3 = 1) represent equal amounts of bound DNA from each amplicon, an indication of no methylation differences between tumor and nontumor genomes. Selection of genes was based on the criteria described in the Materials and Methods. Figures 6(b) and 6(c) showed specific genes of selection results in hypermethylation and hypomethylation, respectively. We conducted a confirmation study to determine whether the cutoff ratio (≧2) could accurately identify hypermethylation. The hierarchical clustering presented the 109 gene loci of hypermethylation in the classifier in N and NA (Figure 2(b)). This methylation profile analysis has led to the identification of CpG island clusters that could evaluate many new genes correlating with OSCC progression in mouse model. These newly collected genes are shown in detail in Table 2. Upon further examination, we selected the* RARB* gene to examine in greater detail by MS-PCR bisulfite sequencing and semiquantitative RT-PCR. The locations of CpG islands in mouse and human* RARB* genes were predicted using MethPrimer (http://www.urogene.org/methprimer/index1.html), respectively (Figures 3(a) and 4(a)).

### 3.3. Verification of Methylated Genes by MS-PCR and Bisulfite Sequencing

The* RARB* gene was a candidate target to verify the methylation status in these mouse OSCC tongues tissues and in human oral cancer cell lines that were also treated by 5′-aza-2′-deoxycytidine (5-aza-dC). Interestingly,* RARB* was hypermethylated in mouse OSCC and reexpression by 5-aza-dC treatment in human oral cancer cell lines. In Figure 3(b),* RARB* was hypermethylated in mouse OSCC tumor parts of N (4-NQO) and NA (4-NQO + Arecoline) compared to normal parts. Furthermore, the methylation ratio of* RARB* in NA treatment at 28 week was 60%. It was much higher than the 4-NQO treatment (20%) at 28 week. These results correlated with the microarray analysis data.* RARB* also investigated the methylation status in human oral cancer lines (Figure 4(b)). The results showed that Ca922, TW2.6, and HSC3 were hypermethylated than in NHOK. It also lost expressions in Ca922, TW2.6, and HSC3 but not in NHOK (Figure 4(c)). In Figure 4(d), the bisulfite sequencing showed the hypermethylation in TW2.6 (92.5%), Ca922 (96.3%), OC2 (90%), and HSC3 (93.8%), but in the NHOK and DOK, the normal and precancer cell lines were not methylated in bisulfite sequencing results. Taken together, these results showed that the promoter methylation of* RARB* plays the main role in OSCC progression.

### 3.4. Methyltransferase Expressions in Human OSCC Cell Lines

**Figure 5:**
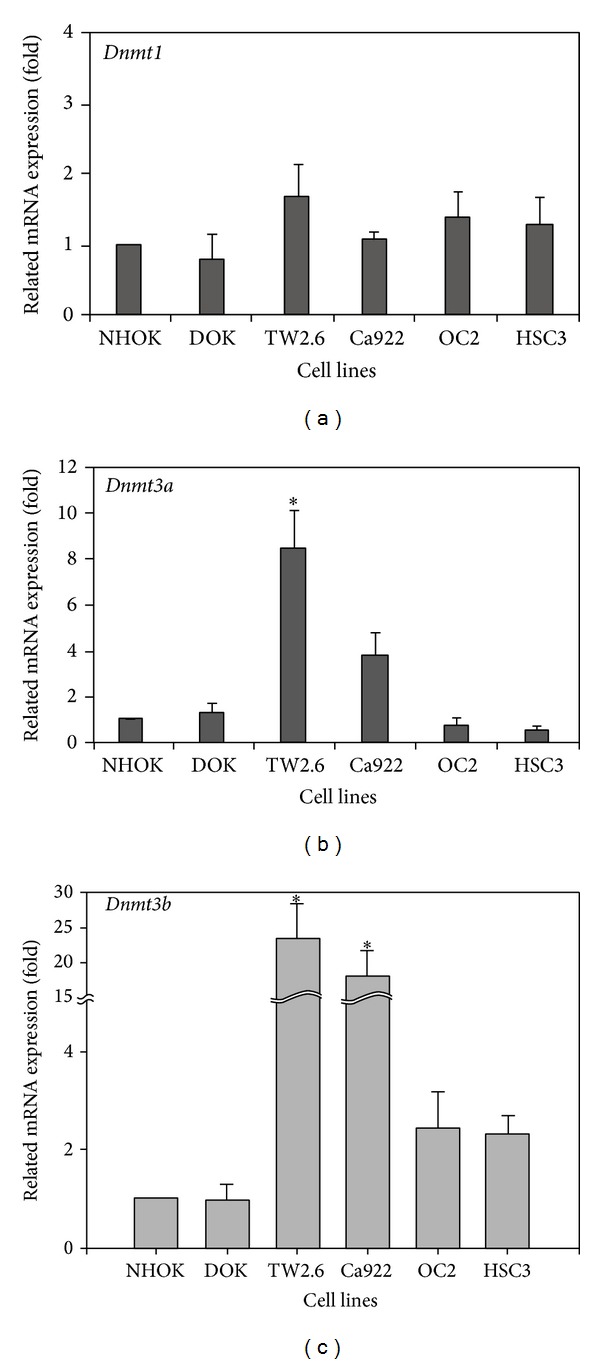
DNMTs RNA expression in human oral cancer cell lines. The measurement of DNMTs expression levels by real-time PCR was showed in (a), (b), and (c), respectively. (a) The* Dnmt1 *expression was not changed in all human oral cancer cell lines. (b) and (c) TW2.6 and Ca922 were higher expression in* Dnmt3a *and* Dnmt3b* than others. The figures shown are the mean of three experiments where all of the samples were analyzed in triplicate. The start sign showed the statistically significant (*P* < 0.05).

**Figure 6:**
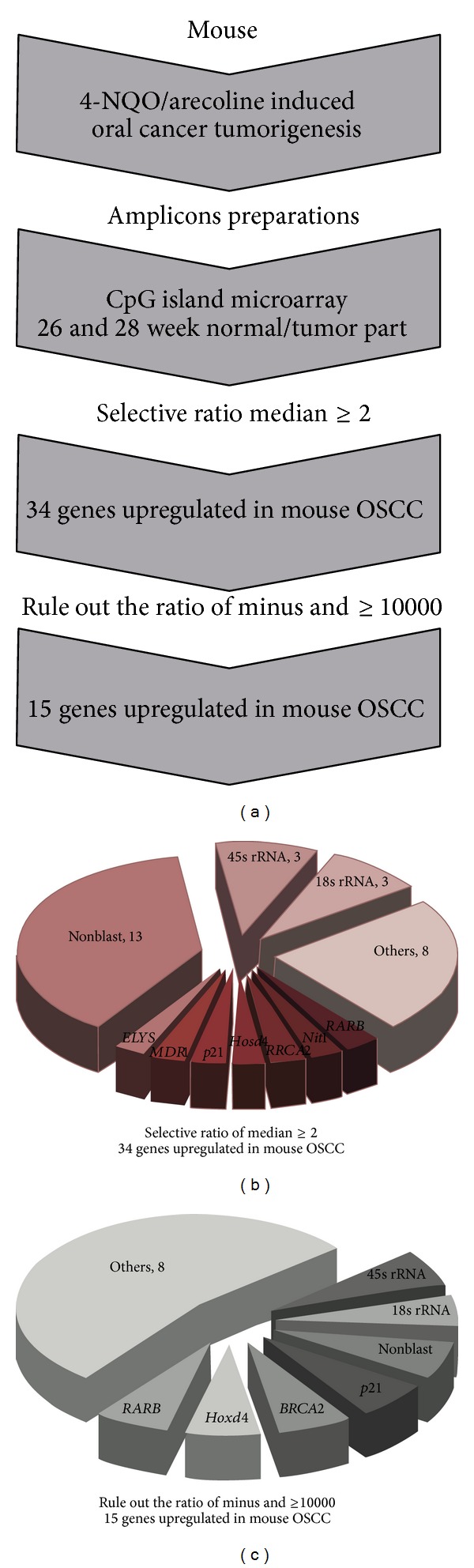
Outline of this study. (a) The flowchart of research project. (b) The selection from MCGI microarray hybridization results by the ratio higher or equal two. There were 34 genes that were hypermethylated in OSCC mouse model. (c) The selection from MCGI microarray hybridization results by ruling out the ratio was minus and higher or equal to 10,000. There were 15 genes that were hypermethylated in OSCC mouse model.

DNA methylation is catalyzed by the family of DNA methyltransferases (DNMT) including* Dnmt1*,* Dnmt3a,* and* Dnmt3b*.  Figure 5 shows the measurement of gene expressions via real-time PCR on* Dnmt1*,* Dnmt3a,* and* Dnmt3b* in human oral cancer lines. For* Dnmt1,* there were no expression differences between NHOK and other cell lines (Figure 5(a)). However,* Dnmt3a* and* Dnmt3b* showed significantly higher expression levels in TW2.6 and Ca922 than others (Figures 5(b) and 5(c)).

## 4. Discussion

OSCC is the most common head and neck neoplasm, affecting 270,000 people worldwide each year [20, 32]. According to related research, patients who smoke, drink, and chew betel quid experience a 5.32-fold increased likelihood of death as compared to those without any oral habits [50]. In Taiwan and other Southeast Asian countries, betel quid chewing is one of the most important risk factors for oral cancer patients and associates as the main cause between betel quid chewing and oral cancer development [51]. 4-NQO is quinoline derivative and a tumorigenic compound which can induce DNA lesions. Quinone oxidoreducatase is one of the major enzymes that convert 4-NQO to the more active metabolite, 3-hydroxyaminoquinoline 1-oxide [52]. This oxidoreducatase can be produced from the mucosal of the sublingual in humans and mice. Arecoline is a natural alkaloid product found in the areca nut. In this study, these two compounds were used to induce oral carcinogenesis in a mouse model. When we added 4-NQO plus Arecoline to the drinking water of mice, the results showed that mice were shown to have induced carcinogenesis at 100% at week 26 and 28 (Figure 1(a)). In H&E staining, resected oral tissue at the end of week 28 (Figure 1(b)) also showed that NA and N treatment could induce squamous cell carcinoma, respectively. However, treatment with arecoline alone revealed that it did not induce tumorigenesis after 28 weeks.

Methylation is important in the development of OSCC and many tumor suppressor genes targeted by promoter methylation will by no doubt be described in the future. The techniques used at present to detect methylation provide good sensitivity, specificity, and speed. There are many types of methylation arrays for a genome-wide approach to realize methylation profile. In this study, we applied a home-made high throughput MCGI array to analyze DNA methylation across the entire genome in the OSCC mouse tongue tissues. This home-made MCGI array not only could identify methylation profile but also could detect mRNA expression (in our previous studies). The chip was cohybridized with mouse Cot-I DNA and total RNA mixture to evaluate the quality and the exon-containing portions can be used to measure levels of gene expression. According to the previous research, the methylation status and mRNA expression levels could be verified by MCGI array.

According to the depicted representative data from previous studies,* RARB* expression is thought to be associated with cellular sensitivity to retinoid in numerous cancer cells, including HNSCC cells, breast cancer cells, lymphocytic leukemia, and lung cancer cells [53–57]. Methylation of* RARB* was identified which had a correlation with primary oral malignant diseases [58, 59]. The methylation array of 4,608 genes (duplicate on chip) that we used in this study included the majority of genes which have previously been associated with head and neck cancer (e.g.,* DAPK*,* MGMT,* and* CDH1*, etc.). However, only* RARB* in previously studied genes were shown to be positive for methylation. To explain the possibility of this discrepancy, the hybridized probes maybe located on different regions between our array and previous methylation studies. However, we found some interesting genes and described this in greater detail in Table 2 as they were shown to have hypermethylation on the chip, but this was not reported before in OSCC.

In our study,* DNMT1* does not affect the expression in human OSCC cell lines. Nevertheless,* DNMT3a* and* DNMT3b* did affect the expressions in human OSCC cell lines (Figure 5). These results showed that* DNMT3a* and* DNMT3b* were higher expressions in TW2.6 and Ca922, the primary oral cancer cell lines, but not in OC2 and HSC3. As compared to other tumors, no correlation was seen between DNMT upregulation and promoter hypermethylation-induced inactivation of tumor-related genes. The exact mechanisms of DNMT upregulation remain unclear, but it is suggested that aberrant DNMT activity, especially with regard to DNMT1, is due to a rapid proliferation of cancer cells because DNMT1 binds to proliferating cell nuclear antigen (PCNA) [60]. Overexpression of all the DNMTs at the mRNA level has been shown for several cancers [45, 61, 62]. However,* DNMT3a* and* DNMT3b* were the* de novo* methyltransferases. In this result (Figures 5(b) and 5(c)), we thought* DNMT3a* and* DNMT3b* were added to new methyl groups to DNA to cause DNA methylation aberrant at the early stage of OSCC progression.

We used some different approaches to deal with the sparseness of data. To begin with we used a methylation array of 4,086 genes (duplicated on chip), which provided more comprehensive data. Here we also suggest that this study could offer a basic evidence that promoter hypermethylation of RARB is correlated with the occurrence of betel-related OSCC.

Isotretinoin (13-cis-retinoic acid) is considered to have the effect of preventing second primary cancers and local or regional recurrence after head and neck cancer is treated. However, in a previous clinical trial, chemoprevention therapy involving retinoid acid does not cause significant differences in early head and neck cancers. The nonsignificant benefit result is due to the small number of patients. The prospective study rendered a small percentage of patients that have second primary cancers and local regional recurrence, thus causing the results to be less significant even though there was trend to have reduced second primary cancers developed and less local regional recurrence in prior studies [63]. However, there was another clinical research using the isotretinoin (13-cis-retinoic acid) (50 to 100 mg per square meter of body-surface area per day) as compared with placebo, to be taken daily for 12 months. They offer significantly reduced second primary cancer development after 32 months of follow-up, and multiple second primary tumors developed in the placebo group [64]. Therefore, they suggest that isotretinoin still has the ability to prevent second primary cancers, but there was less use in preventing the primary site recurrence [64]. There was another human cohort study using an* in situ* hybridization (ISH) analysis checker with 38 pairs of surgical specimens of primary OSCC and noncancerous matched normal control to compare the cellular expression level of RARB. They found the loss of RARB in the advanced OSCCs especially when they are betel quid users [65]. This prominent result also give us an understanding that RARB is really an important factor for chemoprevention for tumor progression. Therefore, betel quid related hypermethylation of RARB will really increase the tumorigenesis and poor treatment outcome of oral cancer. Concerning the mechanisms of the chemoprevention function, there was another study that revealed that the retinoids could suppress basal expression of Cox-2 or EGF-mediated induction of Cox-2 in human oral squamous carcinoma cells [66]. Thus, RARB not only has cell cycle inhibition and tumor suppressant effects, but also anti-inflammatory effects that cease COX-2 related cancerization effects on the oral mucosa.

This is pilot study that talked about the detailed mechanisms of retinoid acid function affected by areca. In the future, we should consider retinoid acid use or RARB related drugs for areca users who are found to have oral tumors or oral cancer to reduce the incidence of oral cancer and to provide a better treatment outcome.

## Figures and Tables

**Table 1:** Primer sets used for RT-PCR, MS-PCR, and bisulfite sequencing.

Primer sets	Sense primer (5′→3′)	Antisense primer (5′→3′)	Tm (°C)	PCR size (bp)
*RARB*-Hu-RT	AGGAGACTTCGAAGCAAG	GTCAAGGGTTCATGTCCTTC	60	771
*Dnmt1*-Hu-RT	TACCTGGACGACCCTGACCTC	CGTTGGCATCAAAGATGGACA	60	102
*Dnmt3a*-Hu-RT	TATTGATGAGCGCACAAGAGAGC	GGGTGTTCCACCCTAACATTGAG	64	110
*Dnmt3b*-Hu-RT	GGCAAGTTCTCCGAGGTCTCTG	TGGTACATGGCTTTTCGATAGGA	62	112
*RARB*-Mo-M	GGATTAGAGTTTTCGTGCGTCG	TACCCCGCCGATACCCAAACG	65	90
*RARB*-Mo-U	GGATTAGAGTTTTTGTGTGTTG	TACCCCACCAATACCCAAACA	62	90
*RARB*-Mo-BS	CCACCCAACTCCATCAAACTC	CCATACAATCAAACATAATCTC	58	476
*RARB*-Hu-M	ATGTCGAGAACGCGAGCGATTC	CTCGACCAATCCAACCGAAACG	64	151
*RARB*-Hu-U	GGATGTTGAGAATGTGAGTGATTT	TACTCAACCAATCCAACCAAAACA	62	155
*RARB*-Hu-BS	GTGTGATAGAAGTAGTAGGAAG	GTGATAGAAGTGGTAGGAAG	55	401

∗Hu: human, Mo: mouse, RT: real-time RT-PCR, M: methylated set, U: unmethylated set, BS: bisulfite sequencing.

**Table 2:** The selected gene list of hypermethylation.

Accession number	Gene symbol	Description	Functions	Related carcinoma types
S80555	*RARB *	Retinoic acid receptor-beta	DNA binding, ligand-dependent nuclear receptor activity	Nonsmall cell lung cancer, hepatoma, bladder cancer, rectal cancer, breast cancer, head and neck squamous cancer
AF069985	*Nit1 *	Nitrilase homolog I	Hydrolase activity	Oral squamous cell carcinoma (in this study)
AL355176	*BRCA2 *	Breast cancer II gene	Protein binding	Bladder cancer, breast cancer, ovarian cancer
AL928664	*Hoxd4 *	Homeo box D4 gene	DNA binding	Breast cancer, leukemia, neuroblastoma
AF457187	*p21 *	Cyclin-dependent kinase inhibitor IA	Cyclin-dependent protein kinase inhibitor activity	Nonsmall cell lung cancer, bladder cancer, breast cancer, ovarian cancer, medulloblastoma, hepatoma
M60348	*MDR1 *	Multidrug-resistance protein gene	ATP binding, ATPase activity	Pancreatic cancer, breast cancer, colorectal cancer, glioblastoma, leukemia, laryngeal cancer cell
AB081498	*ELYS *	Embryonic large molecule derived from yolk sac	DNA binding	Oral squamous cell carcinoma (in this study)
